# Inferring the contribution of small RNAs to changes in gene expression in response to stress

**DOI:** 10.1093/nargab/lqac015

**Published:** 2022-03-04

**Authors:** Meshi Barsheshet, Shira Fisher, Hanah Margalit

**Affiliations:** Department of Microbiology and Molecular Genetics, Institute for Medical Research Israel Canada, Faculty of Medicine, The Hebrew University of Jerusalem, Jerusalem 9112102, Israel; Department of Microbiology and Molecular Genetics, Institute for Medical Research Israel Canada, Faculty of Medicine, The Hebrew University of Jerusalem, Jerusalem 9112102, Israel; Department of Microbiology and Molecular Genetics, Institute for Medical Research Israel Canada, Faculty of Medicine, The Hebrew University of Jerusalem, Jerusalem 9112102, Israel

## Abstract

A main strategy of bacteria adapting to environmental changes is the remodeling of their transcriptome. Changes in the transcript levels of specific genes are due to combined effects of various regulators, including small RNAs (sRNAs). sRNAs are post-transcriptional regulators of gene expression that mainly control translation, but also directly and indirectly affect the levels of their target transcripts. Yet, the relative contribution of an sRNA to the total change in the transcript level of a gene upon an environmental change has not been assessed. We present a design of differential gene expression analysis by RNA-seq that allows extracting the contribution of an sRNA to the total change in the transcript level of each gene in response to an environmental change by fitting a linear model to the data. We exemplify this for the sRNA RyhB in cells growing under iron limitation and show a variation among genes in the relative contribution of RyhB to the change in their transcript level upon iron limitation, from subtle to very substantial. Extracting the relative contribution of an sRNA to the total change in expression of genes is important for understanding the integration of regulation by sRNAs with other regulatory mechanisms in the cell.

## INTRODUCTION

Small RNAs (sRNAs) are major players in the response of bacteria to environmental changes, including stress conditions. These 50–400-nucleotide-long RNA transcripts regulate gene expression post-transcriptionally by base pairing with target transcripts, often in association with a protein chaperone, such as the Hfq protein (1–3). sRNAs act mainly as regulators of target translation, repressing or activating translation initiation by blocking or exposing the ribosome binding site, respectively. In both modes of regulation, the effect of the sRNA on the target’s translation often results in an indirect effect on the mRNA level: translation inhibition is manifested by fewer ribosomes bound to the mRNA, leaving it more exposed to cleavage by endoribonucleases, resulting in a decreased stability and in lower transcript level. Enhanced translation, on the other hand, is manifested by higher ribosome occupancy of the mRNA, protecting it from endoribonucleases, thus increasing its stability and its overall level. Note, however, that in a few studies sRNAs were shown to directly affect the level of their target transcript either by masking, exposing or generating an endoribonuclease binding site [e.g. (4,5)] or by affecting Rho-dependent premature transcription termination [e.g. (6)].

The acknowledgment that sRNAs often affect their target transcript levels has led researchers to use the transcript level as a proxy for the regulatory effect an sRNA has on a target (7). In quite a few studies, the levels of gene transcripts in cells grown under a certain condition were compared between a wild-type strain and a strain that was either deleted of or overexpressing the sRNA [e.g. (8,9)]. Using such comparative studies, the effect of the sRNA on the target expression levels under the studied condition could be obtained. However, such measurements, done under a certain condition, do not provide knowledge about the relative contribution of the sRNA to the change in gene expression when the cell switches from condition A to condition B, including the encountering of a stress condition. sRNAs are only one kind of regulators among many different regulators of gene expression present in a cell at a given moment. A change in growth condition induces the activity of various cellular regulators that control the reprogramming of the transcriptome, increasing the transcript levels of some genes and decreasing the transcript levels of others. The total change in the transcript level of a gene is determined by the combined effects of all its regulators, which may include an sRNA. To understand the effect of the sRNA in the cellular regulatory networks and in the regulation of specific genes upon environmental changes, it is important to extract the contribution of the sRNA to the total change in gene expression upon change in growth conditions. This is conceptually different from merely measuring the change in transcript level following deletion or overexpression of an sRNA in cells grown under the same condition. The experimental–computational approach presented here can provide a reliable representation of the sRNA contribution to the cellular response to environmental changes and expand our understanding of the integration of regulation by sRNAs with other regulatory mechanisms in the cell.

**Figure 1:**
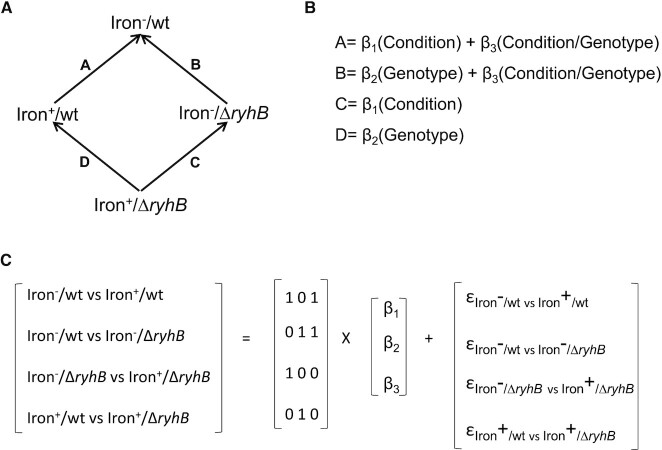
**(A)** Schematic representation of the comparisons of RNA-seq data that were conducted. Two strains (wild type and Δ*ryhB*) were grown to exponential phase, either in LB rich medium containing iron (iron^+^) or under iron limitation induced by treatment with the iron chelator 2,2′-dipyridyl (iron^–^). RNA-seq was applied to total RNA extracted from cells of each condition/strain combination. In each comparison, we computed the log_2_ fold change (log_2_FC) of normalized read counts between the condition/strain combinations at the arrow head and tail. For example, in comparison A, gene expression of the wild-type strain was compared between iron^–^ and iron^+^ and log_2_FC of [normalized read counts (iron^–^)/normalized read counts (iron^+^)] was computed. (**B**) The changes in gene expression in the various comparisons can be written as a linear combination of the changes attributed to the various corresponding factors: the condition effect due to factors independent of RyhB (*β*_1_), the genotype effect independent of condition (*β*_2_) and the joint effect of genotype and condition (*β*_3_). For example, in comparison A, the change in transcript level upon iron limitation (log_2_FC_wt_) is due to the effect of factors independent of RyhB (*β*_1_) and due to the contribution of RyhB (*β*_3_) to the transcript level change. (**C**) We extract *β_i_* by solving the equation system *Y* = *X* × *β* + *ϵ*, where *Y* includes the values of gene expression changes in comparisons A–D, *X* is the design matrix, *β* is the vector of contributions of the three components and *ϵ* is the noise. A multiple linear regression model is used to infer *β_i_* for each gene by minimizing the errors *ϵ* across all conditions and replicates.

Here, we present a straightforward approach to obtain this knowledge, taking advantage of a special design of comparative RNA-seq analyses offered by the DESeq2 package (10), which allows the isolation of the contribution of a specific factor to the change in gene expression upon an environmental change by fitting a linear model to the data (Figure 1). The current availability of large-scale data of direct sRNA targets, which were determined independent of gene expression considerations (11), opens the door to a comprehensive assessment of the sRNA contribution to the total change in the transcript level of its targets upon an environmental change.

We demonstrate this approach by studying changes in gene expression in *Escherichia coli* K-12 MG1655 between cells grown in rich medium and cells grown under iron limitation, focusing on the sRNA RyhB. This sRNA is known to be induced under iron limitation. It upregulates the expression of various genes that are needed under this condition [e.g. (12)] and downregulates the expression of genes encoding proteins that use iron and store it [(8,13); for review, see (14)]. RyhB has been extensively studied in *E. coli* K-12 MG1655, and many of its direct targets were determined experimentally by a variety of large-scale methods, including microarray, RNA-seq, MAPS and RIL-seq, and confirmed by detailed experiments [for review, see (14)]. It was also explicitly shown that in addition to affecting target translation, RyhB affects the transcript levels of many of its targets (8,15,16). However, the relative contribution of RyhB to the change in gene transcript levels in response to iron limitation, which is the aspect that we aim to emphasize in the current study, has not been yet addressed for RyhB (nor for any other sRNA in response to any stress). The total change in the transcript level of a specific gene under iron limitation is determined by the combined effects of all the involved regulators, RyhB being one of them. Using a special design of differential gene expression analysis by RNA-seq presented here, we assess the relative contribution of RyhB to the total change in transcript level a gene undergoes in response to iron limitation (‘sRNA contribution’). Our analysis shows that the relative contribution of RyhB to the change in transcript level varies among genes, and is statistically significant for only a small number of genes, which are enriched with RyhB targets. The experimental/computational approach we present can be applied to any sRNA and environmental condition of interest.

## MATERIALS AND METHODS

### Oligonucleotides and plasmids

All oligonucleotides and plasmids used in this study are listed in Supplementary Table S1.

### Bacterial strains and growth conditions

Bacterial strains used in this study are listed in Supplementary Table S1. As a basis for all experiments and strain constructions, two *E*. *coli* K-12 MG1655 strains were used: (i) a wild-type strain and (ii) a *ryhB* deletion strain (Δ*ryhB*). Bacteria were routinely grown with shaking (200 rpm) at 37°C in LB rich medium. In all experiments, single colonies were suspended in liquid medium and grown overnight. The next day the cultures were diluted 1:100 in fresh medium and grown to an OD_600_ of 0.5. Where indicated, the iron chelator 2,2′-dipyridyl was added to the growth medium (200 μM final) for the last 30 min of growth (resulting in iron limitation).

### RNA extraction

Wild-type and Δ*ryhB* cultures were grown as described earlier, centrifuged for 5 min at 4°C, 4500 × *g*, resuspended in 50 μl TE (10 mM Tris–HCl, pH 7.5, 1 mM EDTA) and mixed with 5 μl of 9 mg/ml lysozyme prior to freezing the sample in liquid nitrogen and storing it at −80°C. Frozen samples were subjected to two cycles of thawing at 37°C and refreezing in liquid nitrogen. Next, the samples were resuspended thoroughly to homogenization with 1 ml TRI Reagent (prewarmed to room temperature) and incubated for 5 min at room temperature. Two hundred microliters of chloroform was added, and the tube content was mixed by inversing the tubes for 15 s. The samples were incubated for 10 min at room temperature, centrifuged (17 000 × *g*, 10 min at 4°C) and the upper phases were collected and transferred into new Eppendorf tubes. For RNA precipitation, 500 μl isopropanol was added, and the tube contents were mixed thoroughly by inversion of the tubes and incubated for 10 min at room temperature. The tubes were centrifuged (17 000 × *g*, 15 min at 4°C) and the supernatant was discarded. The pellets were washed twice by addition of 1 ml of freshly made 75% (vol/vol) ethanol, followed by centrifugation (17 000 × *g*, 5 min at 4°C) and removal of the supernatant. Pellets were air dried at room temperature and then resuspended in 300 μl nuclease-free water and stored at −20°C. The RNA concentration was measured using NanoDrop (Thermo Fisher Scientific).

### Construction of RNA-seq libraries and sequencing

Wild-type and Δ*ryhB* strains were each grown in rich medium containing iron and under iron limiting condition, and RNA was extracted for preparation of RNA-seq libraries. RNA-seq libraries were constructed according to the RNAtag-Seq protocol (17) with several modifications, to enable capturing of short RNA fragments (18). The RNAtag-Seq method enables the inclusion of many RNA samples in a single RNA-seq experiment by barcoding the different samples before library construction. This allows the application of rRNA depletion by RiboZero to all samples together. The libraries were single-end sequenced with read length of 92 bases on NextSeq 500 Sequencer (Illumina). The experiments were performed in six replicates for each strain and growth condition; thus, six libraries were constructed for each combination of condition/strain. After quality and consistency assessments, a subset of the libraries of replicate experiments was maintained for the subsequent analyses (Supplementary Table S2).

### Mapping the sequencing reads to the genome and generation of a count matrix

We used an in-house script to split the reads into their library of origin using the barcode sequences at the beginning of the read. The reads were then processed by cutadapt (19), version 2.10 with default parameters, to remove low-quality ends and adaptor sequences. The fragments were mapped to the genome of *E. coli* K-12 MG1655 (NC_000913.3) using bwa aln followed by bwa samse (20), version 0.7.17-r1188 with default parameters, for single-end sequencing. A custom script was used to retrieve the count files from the bwa files.

### Analysis of differential gene expression using a multiple linear regression

To compare transcript levels between the different libraries, differential expression analysis was performed for each gene using DESeq2 (10), version 1.28.1 (package in R version 4.0.2). The read counts were normalized across all libraries used in the study (listed in Supplementary Table S2). To infer the contribution of the sRNA, DESeq2 was run with the ‘interaction’ design (∼ condition + genotype + condition::genotype), where ‘condition’ refers to the iron status and ‘genotype’ is related to the strain (wild type or Δ*ryhB*). Information on this design can be found in the vignette of DESeq2 (http://bioconductor.org/packages/devel/bioc/vignettes/DESeq2/inst/doc/DESeq2.html#interactions). For each strain, this analysis provides for every gene the log_2_FC of normalized read counts between the two growth conditions differing by their iron status (log_2_FC_wt_ for the wild-type strain and log_2_FC_Δ_*_ryhB_* for the Δ*ryhB* strain). By fitting a linear model to the data (see Figure 1 and the ‘Results and Discussion’ section), which is embedded in the design of the DESeq2 analysis, it is possible to extract the contribution of the different factors affecting the change in gene read counts upon condition change from rich medium to iron limitation. For the wild-type strain, these include the effect due to various factors independent of RyhB (*β*_1_) and the iron-related contribution that involves RyhB (*β*_3_), all in log_2_FC values. *β*_3_ is defined in DESeq2 analysis as the ‘interaction’ term, referring to the joint effect due to condition and genotype (condition::genotype). Also included in the model are gene expression effects that are due to genotype differences (wild type versus Δ*ryhB*, *β*_2_). To determine genes with statistically significant log_2_FC_wt_ and log_2_FC_Δ_*_ryhB_*, we ran DESeq2 with the Wald test. To determine genes with statistically significant log_2_FC of the interaction term, representing the contribution of the sRNA, we ran DESeq2 with the likelihood ratio test. For both tests, the *P*-value was corrected for multiple hypothesis testing by the Benjamini–Hochberg method (21) (termed hereinafter *P*_adj_, for adjusted *P*-value). Results with *P*_adj_ ≤ 0.1 were considered statistically significant.

### Annotating RyhB direct targets

Genes were marked as RyhB direct targets if they were either previously recorded as known targets (14) or identified by RIL-seq as RyhB targets (11). Based on the annotation in RegulonDB (22), genes residing in an operon that includes a reported target of RyhB were marked as such.

### Verification experiments

#### GFP reporter assay

The GFP reporter assays were done essentially as described previously (23,24), using the pXG10-SF as a backbone (23). Wild-type and Δ*ryhB* cells were transformed with a low copy number plasmid expressing the target–GFP fusion (pXG10-SF plasmids). As a control, we used a non-GFP plasmid (pXG0). Single colonies containing either a target–GFP fusion plasmid or a control plasmid were grown overnight, diluted 1:100 in fresh medium and grown with shaking (200 rpm) at 37°C to OD_600_ = 0.5, with or without the addition of chelator 2,2′-dipyridyl. Five hundred microliters of each culture was centrifuged and the pellet was resuspended in 500 μl of 1× PBS. Fluorescence was measured using the BD Accuri™ flow cytometer. Background fluorescence, which was estimated by the fluorescence of cells carrying the control plasmid, was subtracted from the measured fluorescence values. For every strain (wild type and Δ*ryhB*) with a target–GFP fusion, experiments were performed for six biological replicates. The ratios of fluorescence level between the different combinations of strain (wild-type and Δ*ryhB*) and condition (treated and nontreated with chelator 2,2′-dipyridyl) were calculated and the statistical significance was tested by a Mann–Whitney test (two-sided test).

#### qPCR

Bacterial growth and RNA isolation were done as described earlier. Five micrograms of RNA was treated with TURBO DNase (Invitrogen) and used for cDNA synthesis using the SuperScript™ III First-Strand Synthesis System. cDNA was quantified by qPCR using CFX Connect Real-Time PCR Detection System with iTaq™ Universal SYBR Green Supermix (Bio-Rad) in a 96-well plate module according to manufacturer’s instructions. The level of each target gene was tested using specific primer pair and normalized using the level of the *ssrA* RNA. The relative amount of cDNA was calculated by a comparative Ct evaluation method or relative quantification, and ΔΔCt was calculated. The amount of target, normalized to a reference gene (ssrA) and relative to a calibrator (Δ*ryhB*), is given by 2^ΔΔCt^.

#### Western blot analysis

Cultures of wild-type and Δ*ryhB* cells, in which the chromosomal *fur* gene was tagged with a 3x-flag tag, were grown to OD_600_ = 0.5 with/without 2,2′-dipyridyl treatment, collected by centrifugation (4500 × *g*, 10 min at 4°C) and suspended in NP-T buffer (50 mM NaH_2_PO4, 300 mM NaCl, 0.05% Tween 20, pH 8.0). Cells were mixed with 400 μl glass beads and lysed by mixing in a Retch MM400 mixer for 10 min at 30 s^−1^. Cell lysates were cleared by centrifugation (13 000 × *g*, 15 min at 4°C), supernatants were collected and the total protein concentration was measured using NanoDrop. Equal amounts of total protein were mixed with a protein sample buffer, heated at 95°C for 5 min and subjected to a 4–20% polyacrylamide SDS gel electrophoresis followed by electrotransfer onto a nitrocellulose membrane. The membrane was probed with M2 anti-Flag monoclonal antibody (Sigma) and then with anti-mouse secondary antibodies (Jackson ImmunoResearch). Signals were visualized by the ImageQuant LAS 4000 mini system and quantitated using the ImageJ program.

## RESULTS AND DISCUSSION

### Transcriptome experiments measuring the change in gene expression between iron-limited and iron-rich media

We applied RNA-seq to *E. coli* K-12 MG1655 wild-type and Δ*ryhB* strains, each grown to exponential phase in two conditions: (i) growth in rich medium containing sufficient iron and (ii) growth under iron limitation, the latter induced by treating the cells for 30 min with 2,2′-dipyridyl (see the ‘Materials and Methods’ section). We term these conditions iron^+^ and iron^–^, respectively. Thus, there are four types of libraries, defined by the combination of condition/strain: iron^+^/wild type; iron^–^/wild type; iron^+^/Δ*ryhB*; and iron^–^/Δ*ryhB*. Thirteen libraries successfully passed the quality and consistency assessments and they comprised the set of libraries analyzed in the study (Supplementary Table S2). The read counts were normalized by DESeq2 (10) across all 13 libraries.

We first examined the changes in expression levels of RyhB itself and of the transcription regulator Fur, the main regulator of iron homeostasis in *E. coli* and in many other bacteria (25). Under iron-rich conditions, Fe^2+^-bound Fur (Fur-Fe^2+^) becomes active. It mainly acts by repressing the transcription of genes involved in iron uptake and storage in order to prevent unnecessary iron accumulation, but there are some indications that Fur-Fe^2+^ can act also as a transcriptional activator (26). Under iron limitation, Fur is inactive, resulting in derepression of its repressed targets and an increase in their expression levels. One of the targets of Fur-Fe^2+^ is RyhB (13,27). Indeed, we observed in our data high read count corresponding to *ryhB* in the wild-type strain grown in iron^–^ and low read count in the wild-type strain grown in iron^+^ (Supplementary Figure S1). As expected, no reads corresponding to *ryhB* were identified in the Δ*ryhB* strain grown under each of the conditions.

It was suggested that RyhB negatively regulates the translation of *fur* by base pairing with a sequence in the 5′-UTR of an upstream open reading frame coupled in its translation with *fur* (28). Yet, the effect of RyhB on the mRNA and protein levels of *fur* upon induction of iron limitation seemed to be small (28,29) and its physiological implications are not yet known. Our RNA-seq results show no statistically significant difference in the read counts corresponding to *fur* between the four combinations of condition/strain (Supplementary Figure S2). Since it is possible that the effect of RyhB can be identified only at the translation level, we also measured the levels of Fur by western blot analysis. Consistent with the RNA-seq results, no difference was observed in the levels of Fur among the four combinations of condition/strain (Supplementary Figure S2). Thus, it seems that under our experimental conditions the reduced activity of Fur in the iron^–^ condition is mainly due to the lack of iron as its ligand, and not due to reduction in the expression level of Fur. Importantly, this finding suggests that the contribution of RyhB to the change in transcript levels of genes probably does not involve indirect effects through Fur regulation.

**Figure 2:**
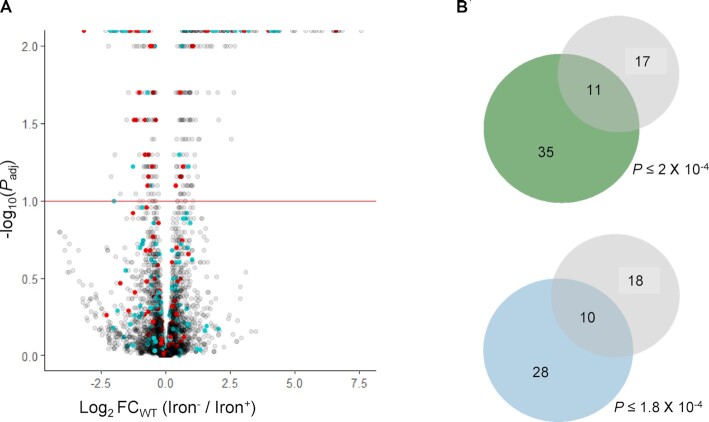
(**A**) A volcano plot of DESeq2 results obtained by comparing RNA-seq data of wild-type *E. coli* K-12 MG1655 strain grown under iron limitation (iron^–^) and in rich medium containing iron (iron^+^) for 4377 genes in the data (comparison A in Figure 1A). Changes in transcript levels are represented by the log_2_FC_wt_, the log_2_ fold change in normalized read counts between the growth conditions, as obtained from DESeq2 analysis (10) (*x*-axis). The statistical significance of the change is represented as −log_10_ *P*_adj_ (*y*-axis). *P*_adj_ is the *P*-value of the log_2_FC value corrected for multiple hypothesis testing. A dashed line is shown for the statistical significance threshold of *P*_adj_ = 0.1 (−log_10_ *P*_adj_ = 1, implying that the dots above this line represent genes with statistically significant changes in read counts). Red dots represent RyhB targets [either previously known (14) or identified by RIL-seq for *E. coli* grown to exponential phase under iron limitation (11)]. Light blue dots represent genes that share an operon with a reported target of RyhB. White dots represent all other genes. (**B)** Venn diagrams of subgroups of genes enriched within the 28 genes for which a statistically significant contribution of RyhB (*β*_3_) to the total change in transcript level was identified (*P*_adj_ ≤ 0.1) (gray). The hypergeometric *P*-value of the enrichment is indicated. Upper panel: previously reported targets of RyhB (green). The *P*-value of the hypergeometric test was computed based on 359 genes for which DESeq2 computed a statistically significant change in transcript level between the growth conditions (Supplementary Table S3), 28 genes with statistically significant *β*_3_ values (RyhB contribution), 46 RyhB targets in the data and 11 targets with statistically significant *β*_3_ values. Lower panel: genes sharing operons with RyhB targets (light blue). The *P*-value of the hypergeometric test was computed based on 359 genes for which DESeq2 computed a statistically significant change in transcript level between the growth conditions, 28 genes with statistically significant *β*_3_ values (RyhB contribution), 38 genes sharing an operon with RyhB targets and 10 genes sharing an operon with RyhB targets with statistically significant *β*_3_ values.

We then turned to examine the genes that changed their expression under iron limitation in the wild-type strain. In total, 359 out of 4377 genes in our data showed statistically significant differences in the normalized read counts between the iron^–^ and iron^+^ conditions in the experiments of the wild-type strain (log_2_FC_wt_ values with *P*_adj_ ≤ 0.1). One hundred fifty genes showed downregulation and 209 genes showed upregulation upon iron stress (Figure 2A and Supplementary Table S3). Among these genes, 46 genes were previously recorded as RyhB targets (11,14), an enrichment that is highly statistically significant by the hypergeometric test (*P* ≤ 1.3 × 10^–11^, based on a total of 4377 genes in the data, 359 genes with *P*_adj_ ≤ 0.1 for log_2_FC_wt_, 195 annotated RyhB targets in the data and 46 targets with *P*_adj_ ≤ 0.1 for log_2_FC_wt_). Also, 38 additional genes reside in operons with annotated RyhB targets (22) (Supplementary Table S3), an enrichment that is also highly statistically significant by the hypergeometric test (*P* ≤ 1.2 × 10^–4^, based on a total of 4377 genes in the data, 359 genes with *P*_adj_ ≤ 0.1 for log_2_FC_wt_, 251 genes residing in operons with RyhB targets, out of which 38 had *P*_adj_ ≤ 0.1 for log_2_FC_wt_).

### Assessing the contribution of RyhB to gene expression change under iron limitation

We used multiple linear regression models to infer the contribution of RyhB to the change in transcript level in response to iron limitation (Figure 1). Briefly, for each gene we computed the log_2_FC between the normalized read counts of RNA extracted from cells grown in the iron^–^ condition and cells grown in the iron^+^ condition, for both the wild-type and the Δ*ryhB* strains (comparisons A and C in Figure 1, respectively). Likewise, we computed the log_2_FC between normalized read counts of RNA extracted from the two strains grown under each condition (comparisons B and D in Figure 1). The log_2_FC_wt_ of each gene, obtained by comparison A, can be described by a linear model that combines the main effect of the condition due to various regulatory factors independent of RyhB (*β*_1_) and the contribution of RyhB (*β*_3_) to the gene expression change (Figure 1B). The latter encompasses the effect of the genotype on the condition change. The log_2_FC obtained by comparison B can be described by a linear model that combines the main effect of the genotype (*β*_2_) and the joint effect of the condition and genotype change (*β*_3_). By fitting a linear model to the data, each of the *β_i_* components can be computed, including the extraction of the contribution of RyhB to the change in gene expression in response to iron limitation (*β*_3_, Figure 1). This linear model (three variables) can be fitted for each gene by minimizing the errors described in Figure 1C, across all replicates (30). To this end, we used a special design of DESeq2 analysis (∼ condition + genotype + condition::genotype), which enables the derivation of the *β_i_* (in log_2_FC values). *β*_3_ is defined in DESeq2 terms as an ‘interaction’ component that distinguishes between the genotypes in their response to the condition change. The inclusion of the ‘interaction’ feature in DESeq2 makes it a natural choice for our purpose, as it is an advanced and highly regarded algorithm developed especially for differential gene expression analysis, which provides statistical significance values for the log_2_FC values (10). For each component, including the interaction term, DESeq2 tests the null hypothesis that the log_2_FC = 0 and assigns a statistical significance, corrected for multiple hypothesis testing by false discovery rate [(21), *P*_adj_ values provided by DESeq2], since we repeat the test for multiple genes. We consider log_2_FC values with *P*_adj_ ≤ 0.1 as statistically significant. Since we focus on the relative contribution of RyhB to gene expression change upon a condition change, in the following discussion we discuss comparisons A and C and the derivation of *β*_1_ and *β*_3_ (Figure 1).

Following the above discussion, we applied DESeq2 to our data and derived for each gene the interaction term, which represents the relative contribution of RyhB to the change in its transcript level upon iron limitation, along with its statistical significance value (Figure 1). These values were extracted for all 4377 genes in our data, and were recorded for the 359 genes included in Supplementary Table S3. Twenty-eight out of the 359 genes have obtained statistically significant log_2_FC values for *β*_3_, the sRNA contribution, 11 of which are previously reported RyhB targets. This enrichment is highly statistically significant by the hypergeometric test (*P* ≤ 2×10^–4^, Figure 2B). Interestingly, no gene outside of the group of 359 genes with statistically significant log_2_FC_wt_ has obtained a statistically significant value for the contribution of RyhB.

### Specific experiments to qualitatively support the relative contribution of RyhB to gene expression change

**Figure 3:**
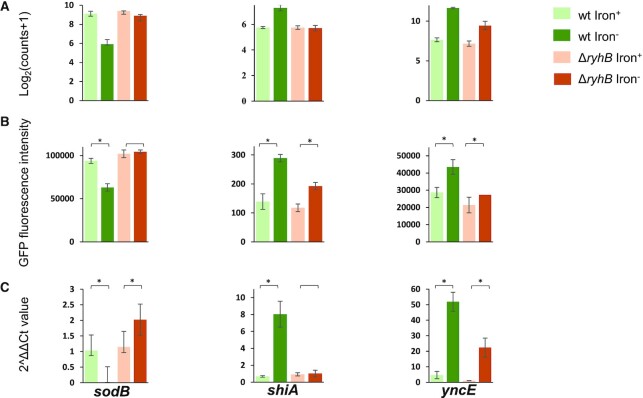
Examples for the contribution of RyhB to the change in expression level of specific genes upon iron limitation. Shown are expression levels of specific genes in the four combinations of condition/strain, measured by various experimental methods. (**A**) Results of RNA-seq experiments expressed in log_2_(read counts + 1). Bars present the mean and standard deviation of a few libraries, as detailed in Supplementary Table S2. (**B**) Results of the GFP reporter assays expressed in fluorescence intensity values. Bars represent the mean fluorescence intensity and 95% confidence intervals of six independent repeats of the GFP reporter assay. (**C**) qPCR results of RNA samples, with amplification performed on a region in the gene’s coding sequence. Bars represent the mean value for 2^ΔΔCt^ after normalization to an endogenous reference and relative to a calibrator. The calculated mean is for three biological replicates with two technical replicates each for every condition/strain combination. The graphs of the same experiment type for different sRNAs are not scaled. The asterisk designates *P* ≤ 0.05 by a two-sided Mann–Whitney *U* test for the indicated comparisons.

**Figure 4:**
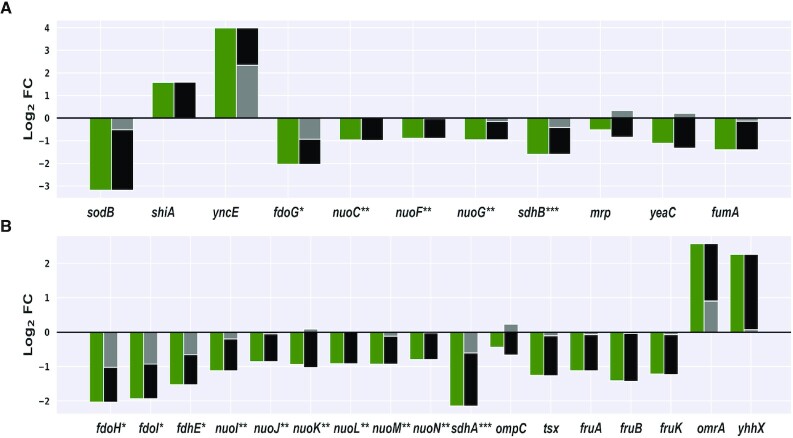
Contribution of RyhB to the change in transcript level of genes. Presented are the log_2_FC_wt_ values representing the total change in transcript level upon iron limitation (green), the component of RyhB contribution to the log_2_FC_wt_ (*β*_3_, black) and the component of the log_2_FC_wt_ that is attributed to factors independent of RyhB (*β*_1_, gray). Shown are genes for which the RyhB contribution to the transcript level change is statistically significant (*P*_adj_ ≤ 0.1). (**A**) RyhB targets. (**B**) Additional genes; some of them share an operon with a RyhB target. Membership in an operon is marked (* for the *fdoGHI-fdhE* operon; ** for the *nuoABCEFGHIJKLMN* operon; *** for the *sdhCDAB-sucABCD-sdhX* operon). Notably, *yhhX*, which is encoded downstream to the *ryhB* gene and is possibly transcribed from *ryhB*’s promoter, showed an increased transcript level in the wild-type strain in the comparison of iron^–^ and iron^+^, and this change was largely attributed to RyhB. This is due to the fact that in the Δ*ryhB* strain the promoter was also deleted.

We selected a few RyhB targets to illuminate the concept of the relative contribution of the sRNA to the change in transcript levels of genes, and to further assess the relative contribution of RyhB by specific experiments (Figure 3): (i) *sodB*: *sodB* encodes the enzyme SodB (superoxide dismutase), which binds iron and destroys superoxide anion radicals that are toxic to the cell (31). Under iron limitation, there is a need to reduce the level of SodB. *sodB* was identified as a target of RyhB and shown to be downregulated by RyhB, which inhibits its translation and enhances the degradation of its mRNA by RNase E (4). Indeed, we obtained by DESeq2 ∼8-fold reduction in the number of reads corresponding to *sodB* in the wild-type strain growing under iron limitation compared to the rich medium condition (log_2_FC_wt_ = −3.19, Figure 3A and Supplementary Table S3), but only a small change in the Δ*ryhB* strain (log_2_FC_Δ_*_ryhB_* = −0.52), which is due to factors independent of RyhB (*β*_1_). The DESeq2 analysis determines a substantial contribution of RyhB to the change in *sodB* expression level (*β*_3_ = −2.67, Supplementary Table S3 and Figure 4), which is highly statistically significant (*P*_adj_ ≈ 0). We verified these results by the GFP reporter assay and qPCR experiments (Figure 3B and C), measuring the expression level of *sodB* in the various combinations of condition/strain at the protein and transcript levels, respectively. The results of DESeq2 analysis, which are qualitatively confirmed by the GFP reporter assay and qPCR experiments, suggest that a major part in the reduction of the transcript level of *sodB* upon iron limitation (∼84%) is due to RyhB, while other factors, independent of RyhB, play a smaller role. (ii) *shiA*: *shiA* encodes a transmembrane transporter of shikimate, which is involved in the shikimate pathway for generating aromatic amino acids. This pathway also produces chorismate, a metabolite that is important for the synthesis of siderophores, which are needed under iron limitation. Thus, *shiA* plays a role in increasing the level of siderophores in the cell under iron limitation (12). RyhB was previously shown to be a positive regulator of the translation of *shiA* and to increase its transcript stability (12). Our results support this conclusion (Figures 3 and 4), where the analysis of the RNA-seq results by DESeq2 indicates that 100% of the increase in transcript level of *shiA* is attributed to RyhB (Figures 3A and 4). The log_2_FC value representing RyhB’s contribution to the change in *shiA*’s transcript level, *β*_3_, is 1.59 (*P*_adj_ ≤ 0.018, Supplementary Table S3), implying it increases *shiA* transcript level ∼3-fold (2^1.59^). The high contribution of RyhB to *shiA* transcript level change is fully supported by the qPCR results (Figure 3C), while the results of the GFP reporter assay suggest that there is a partial increase in ShiA protein expression level even without RyhB, but a greater increase when RyhB is expressed (Figure 3B). (iii) *yncE*: *yncE* was suggested to play a role in the response to iron limitation, as it was found to be upregulated under this condition and a Fur box was identified in its promoter region (32). Thus, *yncE* might be repressed by Fur-Fe^2+^ when iron is prevalent and derepressed and hence upregulated when iron is limited. *YncE* was identified as a target of RyhB in a large-scale screen employing RNA-seq and ribosome profiling, where it was observed to increase in both its transcript level and translation level upon overexpression of RyhB (16). In addition, *yncE* has emerged as one of the major targets of RyhB in a RIL-seq screen of *E. coli* cells grown under iron limitation (11). Our results show that the log_2_FC_wt_ of *yncE* is 3.99 (Supplementary Table S3), implying its transcript level has increased 16-fold (2^3.99^) under iron limitation (Figures 3A and 4). The log_2_FC value of RyhB’s contribution, *β*_3_, is 1.65 (*P*_adj_ ≤ 0.02), suggesting that RyhB contributes only a 3-fold increase (2^1.65^) to the *yncE* transcript level, alluding to a greater effect attributed to the alleviation of the repression by Fur or to other factors independent of RyhB. The relatively small contribution of RyhB to the change in the transcript level of *yncE* is confirmed by the GFP reporter assay, while in the qPCR experiment its contribution seems higher (Figure 3B and C, respectively).

### The contribution of RyhB to transcript level change varies among its targets

The experimental/computational procedure presented here allows to break up the total change in the transcript level of a gene in response to an environmental change into components: a component attributed to the contribution of a regulator of interest (here, RyhB) and a component attributed to factors independent of the regulator of interest. The regulator of interest (here, RyhB) may affect the expression of a gene by direct regulation of the gene and/or by its functional interaction with other regulators of the gene. Thus, the interaction component, *β*_3_, comprises its direct regulation of the target and any indirect effect it might have through its functional interaction with other regulatory factors.

**Figure 5:**
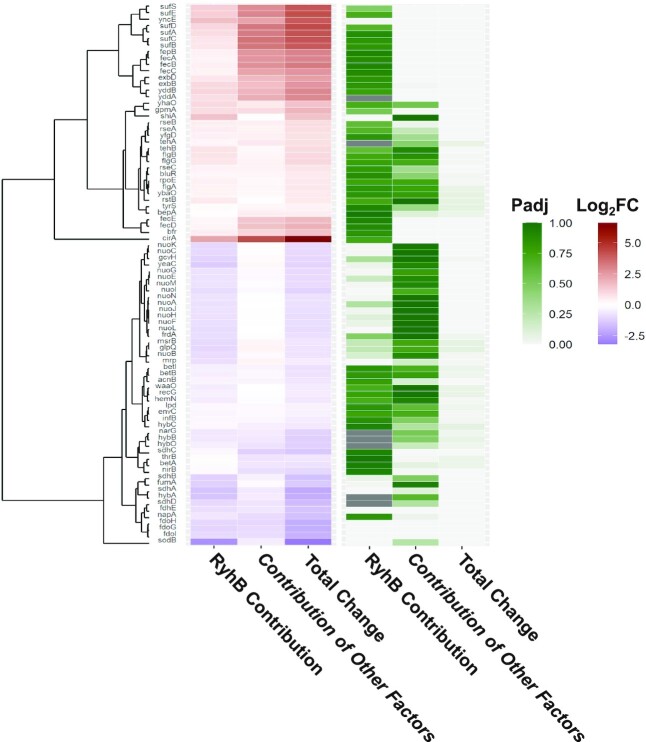
Summary of results for RyhB targets and genes sharing an operon with a RyhB target. Shown are 46 RyhB known targets and 38 genes residing in an operon with a known RyhB target that obtained *P*_adj_ ≤ 0.1 for log_2_FC_wt_. Heatmap (purple to red scale) of the total change in transcript level (log_2_FC_wt_), the contribution of factors independent of RyhB (*β*_1_) and the contribution of RyhB to the total change in transcript level of genes (*β*_3_). The statistical significance values are shown in a white to green scale. Hierarchical clustering was applied to the log_2_FC values by R pheatmap function, using Pearson correlation coefficients between genes.

The derivation of *β*_1_ and *β*_3_ enables assessing whether RyhB and other independent factors affect the total expression change of a gene in the same or opposite directions, opening the door to understand the combination of regulations that sum up to the total expression change observed for a gene. For most of RyhB targets with statistically significant *β*_3_, the parts of the change in transcript level attributed to other independent factors and to RyhB are in the same direction, consistent with the direction of the total change in the transcript level, either upregulation or downregulation (Supplementary Table S3 and Figures 4 and 5). Two exceptions are *mrp* and *yeaC*, where *β*_1_ implies a slight increase in their transcript levels, possibly by positive regulators independent of RyhB, while *β*_3_ implies that RyhB decreased their transcript levels, overcoming the slight increase by the positive regulators and leading to a total decrease in these transcript levels (Figure 4A). For the majority of the downregulated targets in Figure 4A, most of the change in transcript level is attributed to RyhB rather than to possible other independent factors, but these changes are not substantial (*β*_3_ of –1.32 ≤ log_2_FC ≤ –0.8, except for *sodB* with *β*_3_ of –2.67). The two upregulated genes, *shiA* and *yncE*, present a different picture (Figure 4A). First, they vary markedly in the contribution of RyhB to their total increase in transcript levels (100% for *shiA* and only ∼40% contribution for *yncE*). Second, independent of the percentage contribution of RyhB, its absolute contribution to the total increase in the transcript level of these genes seems more substantial than that observed for the downregulated genes (*β*_3_ of 1.59 and 1.65 for *shiA* and *yncE*, respectively).

There were additional 17 genes that exhibited statistically significant *β*_3_ values, which were not recognized before as RyhB targets (Supplementary Table S3, column L, and Figure 4B). Ten of those genes reside in operons with RyhB targets (marked in Figure 4B), and are probably affected by the effect RyhB exerts on their operon member. Indeed, the *β*_3_ values of these genes are very similar to the *β*_3_ values of the targets sharing with them the operon (Supplementary Table S3 and Figure 5). This group of genes sharing operons with RyhB targets is highly enriched among the set of genes with statistically significant *β*_3_ values (*P* ≤ 1.8 × 10^–4^ by the hypergeometric test, Figure 2B). Other genes for which RyhB contributes substantially to their downregulation are genes encoding two porins (*tsx* and *ompC*) and the three genes of the *fruBKA* operon, involved in fructose transport and utilization. While these may be indirect influences of RyhB through other regulators, *fruA* is predicted by the sRNA target prediction algorithm CopraRNA as a target of RyhB (33); thus, *fruB* and *fruK* can be affected by their residence in the operon of *fruA*. Indeed, all three *fru* genes show similar *β*_3_ values.

Interestingly, only 46 out of the 195 previously reported targets of RyhB showed statistically significant changes in their transcript levels under iron limitation (comparison A in Figure 1), and only 11 out of these 46 targets exhibited statistically significant *β*_3_ values (Supplementary Table S3 and Figure 4). Possibly, these targets are affected only at their translation level, without the accompanying effects on their transcript levels. Alternatively, it is possible that our sequencing results do not have the statistical power to detect these transcript level changes and/or the contribution of RyhB to these changes (Supplementary Table S3 and Figure 5). Indeed, 24 of the 46 targets of RyhB in our data had *P* ≤ 0.05, but they failed to pass the significance threshold after the correction for multiple hypothesis testing (Supplementary Table S3). Among them was, for example, *cirA*, previously reported to be directly upregulated by RyhB (34), for which our computations indicated that only a fraction of its total upregulation under iron limitation (log_2_FC_wt_ = 6.6) could be attributed to RyhB (*β*_3_ = 2.29, although with a non-statistically significant *P*_adj_ value). *cirA* is also negatively regulated by Fur-Fe^2+^, suggesting, as in the case of *yncE*, that alleviation of Fur’s negative regulation under iron limitation has a greater effect on *cirA*’s upregulation than the regulation by RyhB. This, once again, emphasizes the importance of isolating the contribution of the sRNA regulator to the transcript level change, which enables understanding its effect on the transcriptome remodeling within the cellular response to the stress.

## CONCLUSION

Disentangling the contributions of various regulators to remodeling the transcriptome is at the basis of a system level understanding of the cellular response to stress. Fitting a linear model to the results of an especially designed framework of comparative transcriptome analysis was shown to be very informative in inferring the contributions of various regulators to such gene expression reprogramming [e.g. (30)]. Here, we applied this approach to isolate the relative contribution of an sRNA to the change in gene expression in response to an environmental change. The isolated contribution of the sRNA comprises its direct effect on the targets and its functional interaction with other regulators. As an example, we demonstrated it for the sRNA RyhB, but it can be performed for any sRNA (or any regulator in general). Also, the approach that we present should be applicable for inferring the relative contribution of an sRNA to the change in translation level upon an environmental change. This can be achieved by applying a similar analysis to ribosome profiling data obtained for the sRNA deletion mutant and wild-type strain in stressed and non-stressed conditions. We believe that extracting the relative contribution of the sRNA out of the total change in expression of a gene provides the basis for elaborate analysis of the dynamics of regulatory circuits combining regulation by sRNAs with other regulators, such as transcription factors (35). Applying the analysis proposed here to RNA-seq or Ribo-seq data of various sRNAs should allow better understanding of their integration in the cellular networks and their contribution to the regulation of the response of bacteria to environmental changes.

## DATA AVAILABILITY

The RNA-seq data reported in this paper can be found in ArrayExpress: E-MTAB-10502.

## Supplementary Material

lqac015_Supplemental_Files
