# Supplementary material for: Inferring the contribution of small RNAs to changes in gene expression in response to stress
Source: NAR Genom Bioinform. 2022 Mar 4;4(1):lqac015. doi: 10.1093/nargab/lqac015 (PMC8896160; doi:10.1093/nargab/lqac015)
Supplement: lqac015_Supplemental_Files [file lqac015_supplemental_files.zip › Supplementary Material-revised.pdf]

Supplementary Material

Inferring the contribution of small RNAs to changes

in gene expression in response to stress

Meshi Barsheshet, Shira Fisher and Hanah Margalit*

Department of Microbiology and Molecular Genetics

Institute for Medical Research Israel-Canada, Faculty of Medicine, The Hebrew University of Jerusalem,

Jerusalem, 9112102, ISRAEL

Supplementary Figures

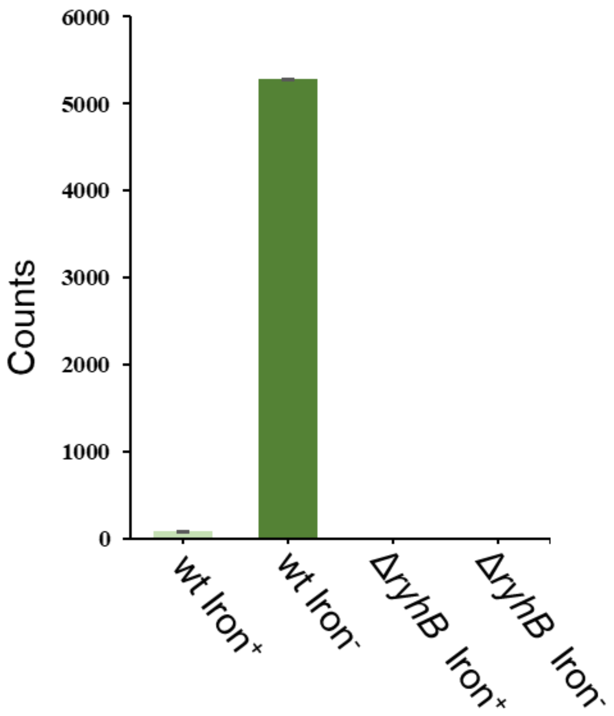

Figure S1: RyhB is induced upon iron limitation

Results of RNA-seq for RyhB in the four condition/strain combinations. The expression level is presented by normalized read counts. Bars present the mean and standard deviation of several libraries (iron^{+}/wild type (light green): four libraries; iron^{-}/wild type (dark green): two libraries; iron^{+}/Δ*ryhB*: three libraries; iron^{-}/Δ*ryhB*: three libraries). RyhB is induced upon iron limitation in the wild type strain, and it is not expressed in the Δ*ryhB* strain.

A

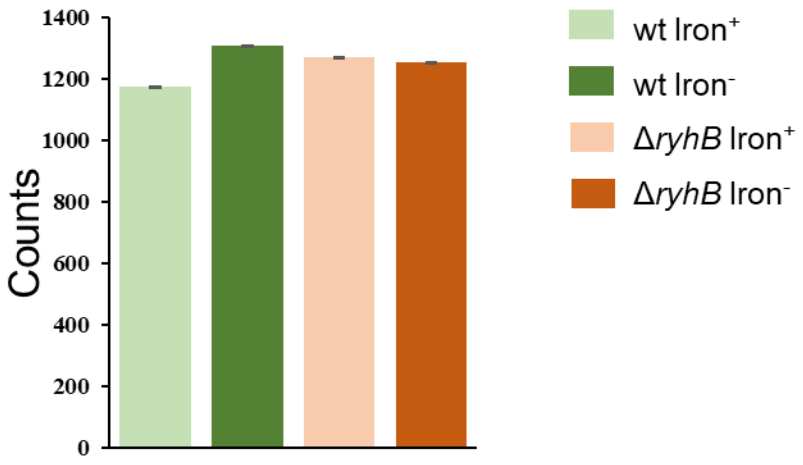

B

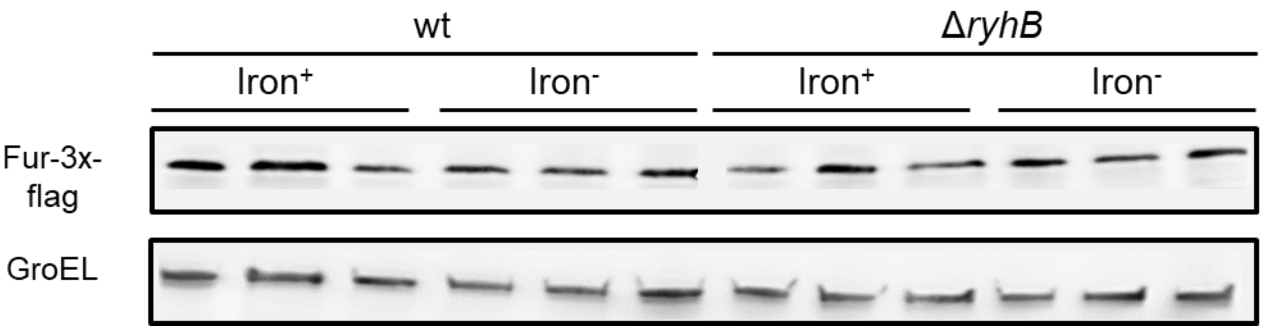

Figure S2: The level of Fur does not change statistically significantly in the four strain/condition combinations

A. Results of RNA-seq for *fur* in the four condition/strain combinations. The expression level is presented by normalized read counts. Bars present the mean and standard deviation of several libraries, as detailed in Figure S1. The slight differences are not statistically significant.

B. Strains in which the chromosomal *fur* is tagged by a 3x-flag tag were constructed on a background of wild type *E.* MG1655 strain (wt) or on a background of Δ*ryhB* strain (Δ*ryhB*). Cultures were grown in rich medium with sufficient iron

(iron^{+}) or under iron^{-}, induced by treatment with the iron chelator 2,2'-Dipyridy (200 µM final; iron^{-}) for 30 minutes before the end of growth (at OD_{600}=0.5). Equal amounts of total protein were analyzed by western blotting using anti Flag antibody. Anti GroEL antibody was used as a loading control.

Supplementary Tables

Supplementary Table S1

List of strains, oligonucleotides and plasmids used in this study

Supplementary Table S2

Supplementary Table S2 - Summary of read counts in sequenced libraries

Supplementary Table S3

Results of DESeq2 analysis and extraction of the relative contribution of RyhB
